# Clinical and instrumental evaluation of non-nutritive sucking in newborns before and after frenotomy: a case report

**DOI:** 10.1590/2317-1782/e20240297en

**Published:** 2025-08-08

**Authors:** Danielle Cristine Marques, Renata Maria Moreira Moraes Furlan, Narciso Sena Fracaroli, Andréa Rodrigues Motta, Estevam Barbosa de Las Casas, Sandra Raquel de Melo Gomes

**Affiliations:** 1 Residência Multiprofissional, Hospital Metropolitano Odilon Behrens - Belo Horizonte (MG), Brasil.; 2 Departamento de Fonoaudiologia, Faculdade de Medicina, Universidade Federal de Minas Gerais – UFMG - Belo Horizonte (MG), Brasil.; 3 Programa de Pós-graduação em Ciências Fonoaudiológicas, Universidade Federal de Minas Gerais – UFMG - Belo Horizonte (MG), Brasil.; 4 Escola de Engenharia, Universidade Federal de Minas Gerais – UFMG - Belo Horizonte (MG), Brasil.; 5 Departamento de Engenharia de Estruturas, Universidade Federal de Minas Gerais – UFMG - Belo Horizonte (MG), Brasil.; 6 Hospital Metropolitano Odilon Behrens - Belo Horizonte (MG), Brasil.

**Keywords:** Sucking Behavior, Infant, Newborn, Ankyloglossia, Breast Feeding, Speech, Language and Hearing Sciences

## Abstract

Changes in the lingual frenulum can impair breastfeeding, leading to poor weight gain and/or early weaning. This study aimed to investigate the influence of frenotomy on the clinical and instrumental parameters of non-nutritive sucking in newborns. It is a case series study with six full-term newborns, three males and three females, diagnosed with ankyloglossia through the Lingual Frenulum Evaluation Protocol for Infants and the Bristol Tongue Assessment Tool. Clinical assessment of non-nutritive sucking was conducted using the Non-Nutritive Sucking Assessment Protocol, and instrumental assessment was performed using an instrument that records sucking pressure. Both assessments were conducted before frenotomy and up to 48 hours after the procedure, respectively, comparing the parameters between these moments. The number of suctions, suction groups, and the mean pressure increased significantly. The evaluation scores also changed significantly after surgery, with a decrease in the lingual frenulum assessment protocol score and an increase in the Bristol Tool score. Instrumental parameters (number of suction groups, total suctions, and mean pressure) and clinical parameters (lip sealing, tongue cupping, tongue dorsum elevation and lowering, mandible elevation and lowering, sucking strength, sucking rhythm, bites, exaggerated mandible excursions, and signs of stress) improved after frenotomy.

## INTRODUCTION

Sucking is a reflex from prenatal stages, starting as early as the 18^th^ week of gestational age (GA), and maturity is reached around 34 to 36 weeks of gestation. It is a fundamental skill for both oral feeding and self-regulation^(1-3)^. Sucking can be classified into two modes: nutritive sucking (NS) and non-nutritive sucking (NNS). NNS is the conditioning of sucking stimuli unrelated to feeding and is attributed some functions, such as reducing stress, reducing pain in hospitalized newborns (NBs), promoting weight gain in premature infants, and gastrointestinal maturation and growth^(4)^. It is characterized by short sucks not associated with swallowing, which, at the end of the process, return to the resting state^(5)^.

Most experts agree that the complexity of mastering the suck-swallow-breathe mechanism is not the only cause of delays in achieving independent oral feeding among hospitalized newborns^(6-8)^. Subjectivity in clinical assessment of bedside sucking may also contribute to delays in achieving oral feeding^(6-8)^. Instruments that assess NNS patterns quantitatively would allow professionals to identify NBs with abnormal patterns early and assess responses to oral interventions objectively^(9,10)^.

A Brazilian instrument for quantitative assessment of infants’ NNS provides information on suction pressure, number of suction groups, number of suctions per group, and duration of pauses^(10)^. The instrument has two parts, a test tip (the part in contact with the baby's mouth) and a vacuum sensor, connected by a flexible, non-collapsible probe. The data are displayed graphically in real time and can be recorded for later analysis. This tool is believed to provide more reliable NNS pattern data for an accurate diagnosis, minimizing the influence of the evaluators' subjectivity and possible contradictions in suction assessment.

Changes in the lingual frenulum can also compromise NBs’ oral feeding. The lingual frenulum is a fold of mucous membrane that connects the tongue to the floor of the mouth^(11)^. It helps stabilize the base of the tongue and does not normally interfere with the movement of the tip of the tongue^(12)^. Ankyloglossia is the complete or partial fusion of the tongue to the floor of the mouth, resulting in limited tongue movement^(13)^. Ankyloglossia can compromise breastfeeding, causing low weight gain and/or early weaning^(14,15)^.

Researchers^(11)^ using the Lingual Frenulum Evaluation Protocol for Infants found that an altered lingual frenulum influences tongue movement during NNS, and the point of attachment of the lingual frenulum influences the rhythm of sucking during breastfeeding. Another study using submental ultrasound showed that the surgical release of the lingual frenulum reduced compression on the maternal nipples, performed by the tongue of NBs with ankyloglossia, improving their breastfeeding performance^(16)^.

More detailed biomechanical research is needed to understand the potential impact on tongue functioning due to anatomical changes in NBs’ frenula, as abnormal ones are believed to impact NBs’ sucking pressure. A method that measures and analyzes this sucking pressure quantitatively may provide data that would help understand the impact of ankyloglossia on tongue mobility and consequent changes in sucking patterns. The present research has not found any other one measuring NBs’ sucking pressure before and after frenotomy.

Thus, this study aimed to verify the influence of lingual frenotomy on clinical and instrumental NNS evaluation parameters in newborns.

## CLINICAL CASE PRESENTATION

This is a case series study with a sample of six NBs, three males and three females, with a mean GA of 39.3 weeks (SD = 3.6) and mean weight at the time of evaluation of 2,658.33 g (SD = 587.97), aged up to 28 days, with evidence of changes in the lingual frenulum, hospitalized in a public hospital between October 2022 and January 2023, in the rooming-in ward at the Conventional Intermediate Care Unit (CInCU) and Kangaroo Neonatal Intermediate Care Unit (KNInCU).

The study was approved by the Research Ethics Committee of the Odilon Behrens Metropolitan Hospital, under approval number 4,480,975 and CAAE 40438420.5.0000.51290. All parents/guardians signed an informed consent form.

The inclusion criteria were full-term birth (over 37 and under 42 weeks of GA); having lingual frenulum changes diagnosed by the Lingual Frenulum Evaluation Protocol for Infants^(11,17,18)^ and the Bristol Tongue Assessment Tool (BTAT)^(19)^; being hospitalized in the CInCU, KNInCU, or rooming-in ward; and not having craniofacial, neuromotor, clinical, or respiratory changes. The exclusion criteria were NBs who did not complete the assessment or had surgery complications.

Participants were subjected to the evaluation procedures explained below.

### Sample characterization (analysis of medical records)

Data were collected from hospital records, including the investigation of the research participants’ previous history: GA, sex, and weight on the assessment date.

### Lingual frenulum evaluation and diagnosis

The same speech-language-hearing pathologist applied the Lingual Frenulum Evaluation Protocol for Infants^(11,17,18)^ and the BTAT^(19)^.

The Lingual Frenulum Evaluation Protocol for Infants^(11,17,18)^ included assessment of lip posture at rest, tongue positioning tendency when crying, raised tongue tip shape when crying, frenulum thickness, frenulum attachment to the sublingual (ventral) surface of the tongue, and frenulum attachment to the floor of the mouth. This study used only the anatomical and functional assessment (part I) of the protocol, which has also been validated as a screening tool^(17)^. The total score ranges from 0 (best result) to 12 (worst result); a frenulum scoring 7 or more is considered abnormal.

The BTAT^(19)^ assesses four aspects of the frenulum: tip of the tongue; attachment of the frenulum to the lower alveolus; elevation of the tongue when crying with the mouth open; and tongue protrusion over the gums. The scores of the four items are added together, ranging from 0 to 8; a frenulum scoring 0 to 3 is considered abnormal.

### NNS clinical evaluation

The NNS clinical assessment was performed using a gloved finger with the baby in supine position (face up), with the neck and head supported and elevated in relation to the rest of the body. The researcher's gloved little finger was inserted between the baby's lips, with the back facing upwards, touching the anterior part of the tongue, gums, and hard palate, and kept in the baby's oral cavity for 3 to 5 minutes. The sucking reflex was considered present when the baby responded with sucking movements alternating with rests. NNS was analyzed for rooting reflex, ease of initiating sucking, lip sealing, tongue cupping, raising and lowering of the tongue dorsum, raising and lowering of the mandible, coordination of lip, tongue and mandible movements, sucking strength, sucking rhythm, biting, exaggerated mandible excursions, and signs of stress, as determined by the NNS Assessment Protocol^(20)^ designed for premature babies. Each protocol item receives a score, and the baby can have a total score from 21 to 86. The higher the score, the better the baby's NNS performance.

### NNS instrumental assessment

The equipment used in this study was developed by researchers from the Biomechanical Engineering Research Group at the Federal University of Minas Gerais, Brazil^(10)^. The method simulates NNS clinical assessment in NBs in speech-language-hearing practice. The device has two distinct parts, connected by a flexible and non-collapsible probe, namely: test tip – formed by three elements: central body, intermediate body, and sealing capsule. The central body, made of non-toxic plastic material, connects the sensor (through the polyethylene tube) with the parts in direct contact with the NB, capturing the suction. The intermediate body is in direct contact with the NB's mouth. It is disposable, made of non-toxic silicone for individual use, and changed for each participant. The sealing capsule is a piece of non-toxic silicone that attaches the intermediate body to the central body so that air does not escape. The instrument uses a vacuum sensor that captures negative pressure and generates a signal that, when properly treated, is digitally transmitted, processed, and stored. The results are displayed in real time on a computer. Computerized treatment systematized the variables to analyze the records, namely: 4.5 Hz frequency filter; auxiliary curve to identify peaks, beginning, and end of suction groups; average thresholds; and minimum pause criterion of 3 s.

**Figure 1:**
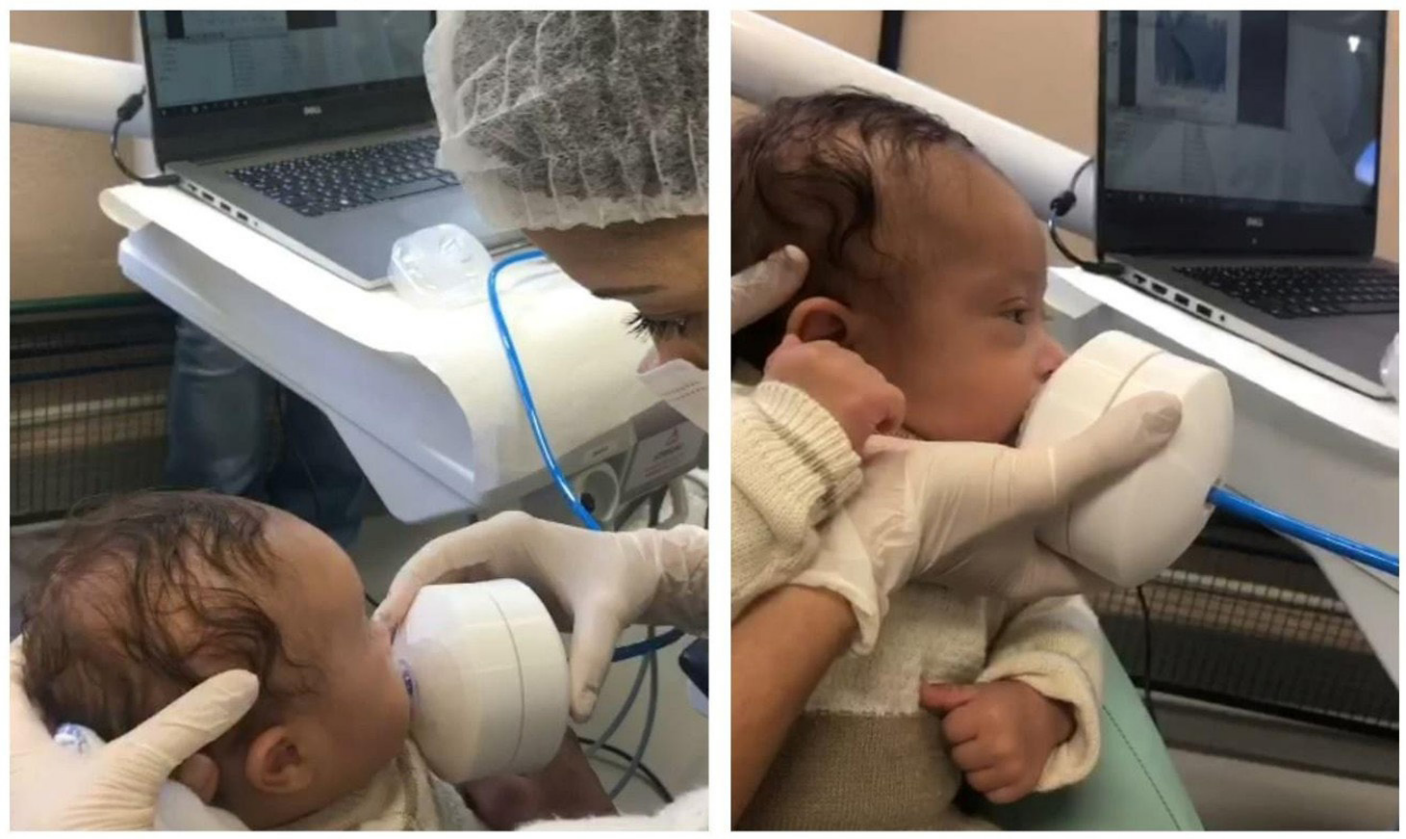
Representative image of the instrumental tongue assessment

For evaluation, the test tip was inserted between the NB's lips, touching the front of the tongue, gums, and hard palate, capturing suction pressure (Figure 1). Two measurements were taken for each NB, each test lasting 2 minutes, with a 2-minute interval between measurements.

This study characterized objective suction pattern measures by defining the following suction behavior parameters for extraction and analysis:

- Number of sucking groups: These were characterized by three or more sucks with intervals between them lasting less than 3 seconds;
- Number of suctions: Number of suctions in the tracing that make up suction groups;
- Mean time of pauses: Mean length of pause intervals;
- Mean pressure value: Mean of the group pressure peaks (kPa).

All six NBs underwent lingual frenotomy performed by the same pediatric surgery team, with three surgeons. They followed the same protocol, using scissors for all cases. Clinical and instrumental suction assessments were performed before frenotomy and up to 48 hours after the procedure (the clinical followed by the instrumental assessment), with an average collection time of about 20 minutes.

### Data analysis

A descriptive analysis of the frequency distribution of all categorical variables was performed, as well as an analysis of the measures of central tendency and dispersion of continuous variables. A comparison analysis between the exposure variables under study and the events was also performed using Student's paired t-test for dependent samples with normal distribution and the Wilcoxon test for dependent samples with non-normal distribution. The Shapiro-Wilk test was applied to define data normality, and the Friedman test was used to correlate the study variables. Results with a 5% significance level were considered statistically significant associations, and the Statistical Package for the Social Sciences (SPSS), version 25.0, was used for input, processing, and analysis of quantitative data.

## DISCUSSION

Table 1 presents information on the NBs’ characteristics regarding GA, weight, hospitalization sectors, and frenulum evaluation scores. The Lingual Frenulum Evaluation Protocol for Infants scores decreased, and the BTAT scores increased after surgery. The mean NNS clinical evaluation score before frenotomy was 39.8 in the positive items and 15.6 in the negative items; after frenotomy, it was 77.3 in the positive items and 3.1 in the negative items.

**Table 1:** Descriptive measures of sample variables

**Explanatory Variables**		**n**	**%**	**µ (±SD)**
**Gestational age**	37 weeks	3	50	39.3 (±3.6)
	38 weeks	1	16.7	
	40 weeks	2	33.3	
**Weight**	≥ 1,500 g and ≤ 2,500 g	3	50	2,658.33 (±587.97)
	> 2,500 g and ≤ 3,000 g	0	0	
	> 3,000 g	3	50	
**Lingual Frenulum Assessment Score**	Bristol Tool – before frenotomy	6	100	3 (±2.4)
	Bristol Tool – after frenotomy	6	100	6.3 (±0.8)
	Lingual Frenulum Evaluation Protocol for Infants – before frenotomy	6	100	8 (±1.3)
	Lingual Frenulum Evaluation Protocol for Infants – after frenotomy	6	100	4.2 (±1.9)
**Sectors**	CInCU	2	33.3	-
	KNInCU	1	16,6	
	Rooming-in ward	3	50	

**Caption:** n = number of participants; % = valid percentage; µ = mean; SD = standard deviation; CInCU = Conventional Intermediate Care Unit; KNInCU= Kangaroo Neonatal Intermediate Care Unit

**Figure 2:**
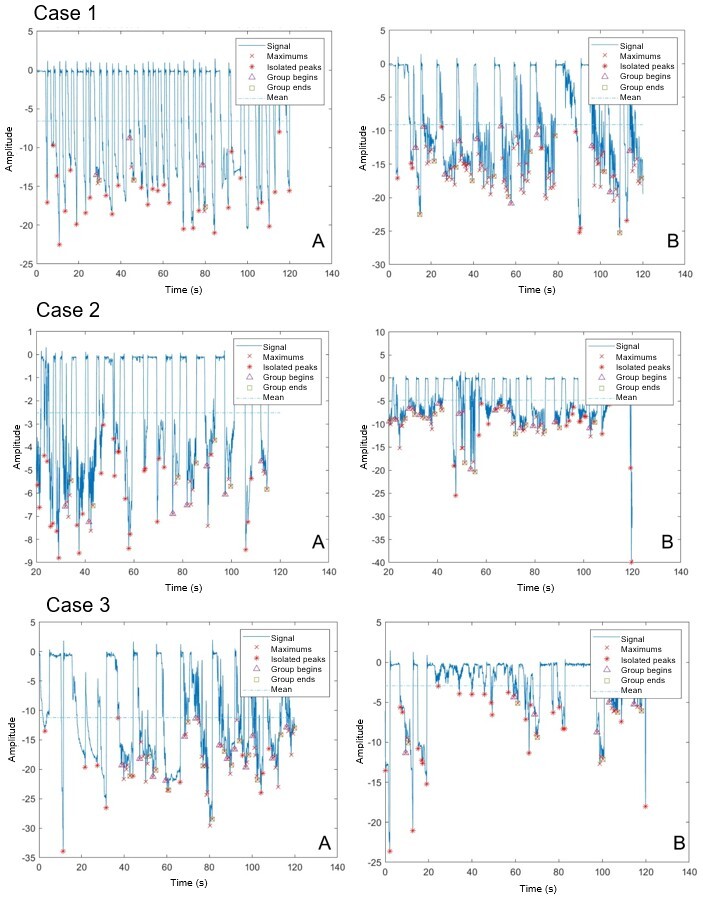
Graphs of instrumental assessment of non-nutritive sucking before (A) and after (B) frenotomy – Cases 1 to 3

**Figure 3:**
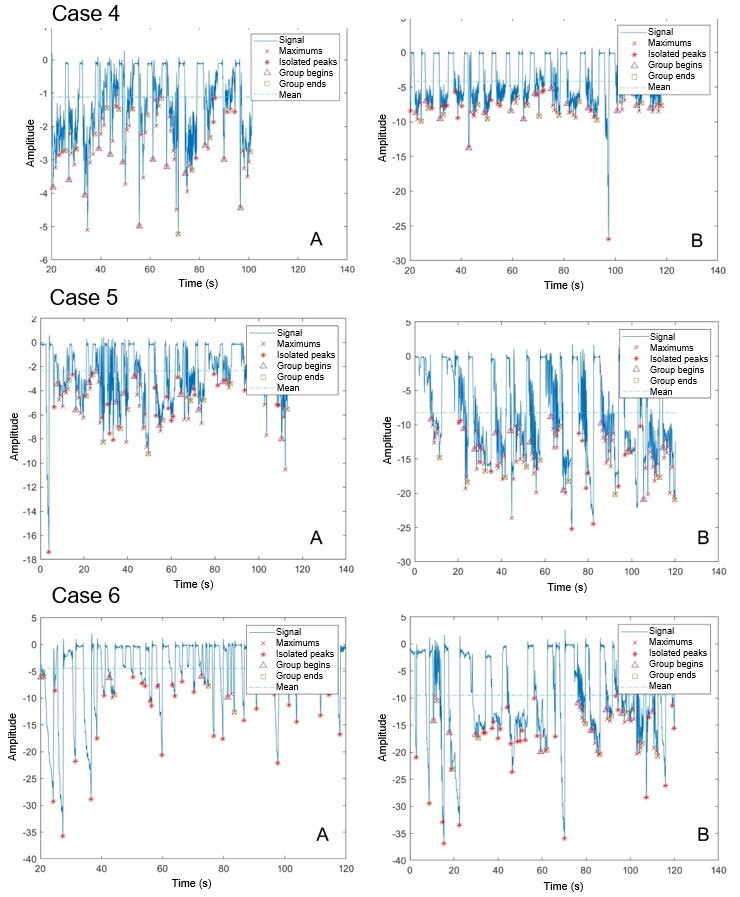
Graphs of instrumental assessment of non-nutritive sucking before (A) and after (B) frenotomy – Cases 4 to 6

Figures 2 and 3 show the tracings of the six NBs’ NNS instrumental evaluation. The number of suctions and the number of groups increased significantly, and the mean pressure also increased.

Table 2 shows the comparison of measurements of the NNS instrumental assessment before and after frenotomy. The number of suction groups, total number of suctions, and mean pressure were statistically significantly different.

**Table 2:** Comparison of objective measures of instrumental assessment of non-nutritive sucking before and after frenotomy

**Variables (Parameters)**	**n**	**Before frenotomy (µ)**	**After frenotomy (µ)**	**p-value**
**No. of suction groups**	6	7.83	12.50	0.028^1^*
**Total no. of suctions**	6	36.5	70.8	0.045^2^*
**Mean no. of suctions per group**	6	4.19	5.76	0.162^2^
**Mean time of pauses (s)**	6	13.95	5.18	0.068^2^
**Mean pressure (kPa)**	6	-7.03	-13.78	0.006^2 *^

1 Wilcoxon test;

2 Paired t-test

* Statistically significant results with p-value ≤ 0.05

**Caption:** no. = number; n = number of participants; s = seconds; kPa = kilopascal; µ = mean

The descriptive analysis of the topics of the NNS Assessment Protocol revealed that all NBs had a rooting reflex and easily initiated sucking, regardless of lingual frenulum changes. However, all other aspects were affected, as observed by comparing the positive items (which increased) and negative items (which decreased) before and after frenotomy in the group. Moreover, 10 of the 12 items were statistically significantly associated, namely: lip sealing, tongue cupping, tongue dorsum elevation and lowering, mandible elevation and lowering, lip coordination, tongue and mandible movements, suction force, suction rhythm, biting, exaggerated mandible excursions, and signs of stress (Table 3).

**Table 3:** Comparison of clinical evaluation parameters of non-nutritive sucking of paired samples before and after frenotomy

**Variables**		n	**Before frenotomy (µ)**	**After frenotomy (µ)**	**p-value**
POSITIVE	Rooting reflex	6	4	4	0
	Suction starts easily	6	2.67	4	0.346^1^
	Lip sealing	6	4.83	8.83	0.039^2^*
	Tongue cupping	6	3	7.5	0.031^1^*
	Tongue dorsum elevation and lowering	6	4.5	7.5	0.012^2^*
	Mandible elevation and lowering	6	4.83	7.5	0.048^1^*
	Coordination of lip, tongue, and mandible movements	6	6.5	12.5	0.026^1 *^
	Suction force	6	4.67	10	0.031^1^*
	Suction rhythm	6	3.33	9.67	0.008^2^*
NEGATIVE	Biting	6	-2.16	-0.33	0.002^2^*
	Exaggerated mandible excursions	6	-2.66	0.66	0.001^2^*
	Signs of stress	6	-10.83	-2.5	0.034^1^*

1 Wilcoxon test;

2 Paired t-test

* Statistically significant results with p-value ≤ 0.05

**Caption:** n = number of participants; µ = mean

This research’s main finding is that the clinical and instrumental NNS parameters improved significantly after lingual frenotomy.

Typical NNS occurs at a rate of two suctions per second, twice as frequent as NS^(6)^. A North American study^(21)^ used an instrumental assessment called NeoSuck Assessment of the NTrainer System to quantify factors related to NNS performance. It evaluated groups of suctions per minute, number of suctions in 1 minute, and peak pressure during suction^(21)^, corroborating the findings of the present study, as it found a mean of 4 to 6 suctions per group.

The literature^(11,16)^ states that lingual frenulum changes can impact suction. This was evidenced in the present study, since patients had better suction group organization and distribution and greater signal amplitude after frenotomy, showing increased suction pressure.

Using an artificial nipple with small sensors, researchers^(22)^ measured the force applied to the nipple by the tongue of healthy infants and infants with difficulty sucking due to ankyloglossia. There was a statistically significant difference between the maximum force measured at the tip and base of the nipple and the difference in time required to reach maximum forces in the groups with and without changes. This study found similar results, with increased mean pressure after frenotomy, approximately 5 kPa. Also, the mean pause time decreased after the procedure, contributing to a better sucking rhythm, although with no statistically significant result.

Another study^(23)^ found lower electrical activity of the suprahyoid muscles during breast sucking in infants with the frenulum fixed at the tip of the tongue, regardless of age. This result indicates that anatomical characteristics of the lingual frenulum may reduce muscle activity due to restricted movement of the tip of the tongue during sucking. It also corroborates that abnormal frenula impact the activity of the suprahyoid muscles, creating compensatory movements. The present study found that mandible elevation and lowering, exaggerated mandibular excursion, biting, and signs of stress improved or disappeared after frenotomy, improving NBs’ sucking pattern and eliminating compensatory facial and mandibular movements in the NNS clinical evaluation.

Lingual frenulum assessment is mandatory^(11)^ and is part of NBs’ physical examination. However, there is controversy among health professionals regarding abnormal lingual frenulum classification. Most diagnose ankyloglossia with subjective criteria, correlated with clinical findings. Lingual frenulum must be assessed with a clinical protocol to help professionals reach the correct diagnosis and prescribe the appropriate treatment.

The Brazilian Ministry of Health recently recommended that trained professionals in the health team use the BTAT^(19)^, and federal law indicates and recommends using the Lingual Frenulum Evaluation Protocol for Infants in the Neonatal Tongue Screening Test, which is mandatory in maternity wards and hospitals since 2014. In clinical practice, each service uses the most adaptable and appropriate protocol for the scenario; hence, it was decided to apply both in this research.

This study found that the scores of the protocols used for measurement changed after frenotomy, showing functional and anatomical gains in tongue mobility. Surgical release of ankyloglossia through frenotomy can correct the restriction of tongue movement during feeding to allow more effective breastfeeding and reduce maternal nipple pain caused by reduced friction between the child's gum/lower tongue and the nipple^(24)^. In this study, all NBs had difficulty maintaining the latch before frenotomy, and in three cases (1, 4, and 6), the mothers had nipple fissures.

A study^(25)^ examined the influence of frenotomy in NBs younger than 12 weeks with posterior ankyloglossia, quantifying changes in breastfeeding and maternal nipple pain using the LATCH instrument. It found that the LATCH score improved significantly immediately after frenotomy. This study did not assess the functioning and effectiveness of frenotomy during breastfeeding. However, NNS objective assessment data suggest that frenotomy brought benefits to these NBs’ sucking pattern.

According to the results of the study by Martinelli et al.^(26)^, the number of sucks increased and the duration of pauses between NS groups decreased after frenotomy in babies with ankyloglossia. Moreover, the mothers' complaints decreased regarding baby's tiredness when breastfeeding, long pauses between sucks, short intervals between feedings, short periods of sleep between feedings, nipple biting pattern, noises during breastfeeding, nipple pain, regurgitation, and coughing. The present study analyzed only the NNS but also showed an improved sucking pattern after frenotomy, as there was an average increase of approximately five suck groups and 35 sucks after the procedure, besides an increased mean pressure of approximately 6 kPa, and a decreased mean pause time by approximately 9 seconds. The NNS clinical assessment found that the suction strength increased by approximately 5 points, and the rhythm improved. Concerning negative points, the scores on bites and signs of stress decreased by approximately 2 and 8 points, respectively.

Future research should conduct a long-term follow-up of participants and include an NS assessment to verify the predictors and the effect of the intervention in resolving possible breastfeeding difficulties. The few participants and the lack of evaluator blinding are limitations of the present study. Nonetheless, it pioneered in presenting quantitative NNS measures and comparative analysis of these measures in individuals before and after frenotomy.

## FINAL CONSIDERATIONS

This study showed considerable improvement in NNS patterns in babies undergoing lingual frenotomy, considering instrumental measures (number of sucking groups, number of total suctions, and mean pressure) and clinical measures (lip sealing, cupping, tongue dorsum elevation and lowering, mandible elevation and lowering, sucking force, sucking rhythm, biting, exaggerated mandible excursions, and signs of stress), with favorable effects on lingual movement functioning.
