# Supplementary material for: Clinical and instrumental evaluation of non-nutritive sucking in newborns before and after frenotomy: a case report
Source: Codas. 2025 Aug 8;37(4):e20240297. doi: 10.1590/2317-1782/e20240297en (PMC12337714; doi:10.1590/2317-1782/e20240297en)
Supplement: Translation

# Translation: Avaliação clínica e instrumental da sucção não nutritiva em recém-nascidos antes e após frenotomia: relato de casos

**DOI:** 10.1590/2317-1782/e20240297pt

**Authors:** Danielle Cristine Marques, Renata Maria Moreira Moraes Furlan, Narciso Sena Fracaroli, Andréa Rodrigues Motta, Estevam Barbosa de Las Casas, Sandra Raquel de Melo Gomes

**Affiliations:** 1 Residência Multiprofissional, Hospital Metropolitano Odilon Behrens - Belo Horizonte (MG), Brasil.; 2 Departamento de Fonoaudiologia, Faculdade de Medicina, Universidade Federal de Minas Gerais – UFMG - Belo Horizonte (MG), Brasil.; 3 Programa de Pós-graduação em Ciências Fonoaudiológicas, Universidade Federal de Minas Gerais – UFMG - Belo Horizonte (MG), Brasil.; 4 Escola de Engenharia, Universidade Federal de Minas Gerais – UFMG - Belo Horizonte (MG), Brasil.; 5 Departamento de Engenharia de Estruturas, Universidade Federal de Minas Gerais – UFMG - Belo Horizonte (MG), Brasil.; 6 Hospital Metropolitano Odilon Behrens - Belo Horizonte (MG), Brasil.

**Keywords:** Comportamento de Sucção, Recém-nascido, Anquiloglossia, Aleitamento Materno, Fonoaudiologia

## Abstract

Alterações do frênulo lingual podem comprometer a amamentação, causando baixo ganho de peso e/ou desmame precoce. O objetivo deste estudo foi verificar a influência da frenotomia nos parâmetros da avaliação clínica e instrumental da sucção não-nutritiva em recém-nascidos. Trata-se de um estudo de série de casos, com seis recém-nascidos a termo, três do sexo masculino e três do sexo feminino, com anquiloglossia, diagnosticada utilizando-se o Protocolo de Avaliação do Frênulo da Língua em Bebês e Protocolo Bristol de Avaliação da Língua. A avaliação clínica foi realizada por meio do Protocolo da Avaliação da Sucção Não-Nutritiva, e a avaliação instrumental utilizando um equipamento que registra a pressão de sucção. Ambas foram realizadas antes da frenotomia e até 48h após o procedimento, respectivamente, sendo realizada a comparação dos parâmetros entre esses momentos. Foi possível verificar aumento significativo da quantidade de sucções, de grupos de sucções e da pressão média. Houve mudança significativa na pontuação das avaliações após realização do procedimento cirúrgico, com diminuição do escore no protocolo de avaliação do frênulo lingual e aumento no protocolo Bristol. Os parâmetros instrumentais (número de grupos de sucções, número de sucções totais, pressão média) e clínicos (vedamento labial, canolamento, elevação e abaixamento de dorso de língua, elevação e abaixamento de mandíbula, força de sucção, ritmo de sucção, mordidas, excursões exageradas de mandíbula, sinais de estresse) de sucção melhoraram após frenotomia.

## INTRODUÇÃO

A sucção surge como um reflexo desde as fases pré-natais, iniciando-se já na 18ª semana de idade gestacional, sendo que a maturidade é alcançada por volta de 34 a 36 semanas de gestação. É uma habilidade fundamental, tanto para a alimentação oral quanto para a autorregulação^(1-3)^. A sucção pode ser classificada em dois modos: sucção nutritiva (SN) e sucção não-nutritiva (SNN). A SNN é o condicionamento dos estímulos de sucção não relacionados à alimentação, sendo a ela atribuídas algumas funções, como redução do estresse, redução da dor no recém-nascido (RN) hospitalizado, promoção de ganho de peso no prematuro, maturação e crescimento gastrointestinal^(4)^. É caracterizada pela presença de sucções curtas, não associadas à deglutição, que, ao final do processo, retornam ao estado de repouso^(5)^.

A maioria dos especialistas concorda que a complexidade de dominar o mecanismo de sucção-deglutição-respiração não é a única causa de atraso na obtenção de alimentação oral independente entre RNs hospitalizados^(6-8)^. A subjetividade na avaliação clínica da sucção à beira leito também pode contribuir para atrasos na obtenção de alimentação oral^(6-8)^. Com instrumentos para avaliar quantitativamente os padrões de SNN, os profissionais poderiam identificar precocemente RNs com padrões anormais e avaliar objetivamente as respostas às intervenções orais^(9,10)^.

Um instrumento para avaliação quantitativa da SNN de bebês, desenvolvido no Brasil, fornece informações sobre a pressão de sucção, número de grupos de sucção, quantidade de sucções por grupo e duração das pausas^(10)^. O instrumento é constituído por duas partes, uma ponta probatória (parte em contato com a boca do bebê) e um sensor de vácuo, conectados entre si por uma sonda flexível e não colabável. Os dados são visualizados graficamente em tempo real e podem ser gravados para posterior análise. Acredita-se que essa é uma ferramenta que pode fornecer dados mais fidedignos do padrão de SNN, para um diagnóstico preciso, minimizando a influência da subjetividade dos avaliadores e, consequentemente, possíveis contradições no que se refere à avaliação da sucção.

Outro fator que pode comprometer a alimentação por via oral de RNs é a alteração do frênulo lingual. O frênulo lingual é uma prega de membrana mucosa que conecta a língua ao assoalho da boca^(11)^. Ajuda a estabilizar a base da língua e normalmente não interfere no movimento da ponta da língua^(12)^. Anquiloglossia é a fusão completa, ou parcial da língua no assoalho da boca, culminando no movimento limitado da língua^(13)^. A anquiloglossia pode comprometer a amamentação, causando baixo ganho de peso e/ou desmame precoce^(14,15)^.

Utilizando o Protocolo de Avaliação do Frênulo da Língua em Bebês, pesquisadores^(11)^ verificaram que o frênulo lingual alterado influencia o movimento da língua durante a SNN e o ponto de fixação do frênulo na língua influencia o ritmo da sucção durante a amamentação. Outro estudo, utilizando ultrassom submentual, evidenciou que o procedimento de liberação cirúrgica do frênulo lingual diminuiu a compressão nos mamilos maternos, realizada pela língua de RNs com anquiloglossia, melhorando o desempenho dos mesmos na amamentação^(16)^.

Pesquisas biomecânicas mais detalhadas são necessárias para entender o impacto potencial na funcionalidade da língua em decorrência de alteração anatômica do frênulo em RNs. Acredita-se que o frênulo lingual alterado pode impactar na pressão de sucção realizada pelo RN e um método que meça e analise quantitativamente essa pressão de sucção fornecerá dados que auxiliarão na compreensão do impacto da anquiloglossia na mobilidade de língua e conseguinte alteração no padrão de sucção. Nenhum estudo que mensurasse a pressão de sucção em RNs pré e pós-frenotomia foi encontrado.

Dessa forma, o presente estudo tem como objetivo verificar a influência da frenotomia lingual nos parâmetros da avaliação clínica e instrumental da sucção não-nutritiva em recém-nascidos.

## APRESENTAÇÃO DO CASO CLÍNICO

Trata-se de um estudo de série de casos, com amostra composta por seis recém-nascidos, três do sexo masculino e três do sexo feminino, com média de idade gestacional de 39,3 semanas (DP=3,6) e média de peso no momento da avaliação de 2.658,33 g (DP=587,97), tempo de vida de até 28 dias, com evidências de alterações no frênulo lingual e que estavam internados em um hospital público entre os meses de outubro de 2022 e janeiro de 2023, nos setores Alojamento Conjunto, Unidade de Cuidados Intermediários Convencional (UCINCo) e Unidade de Cuidado Intermediário Neonatal Canguru (UCINCa).

O estudo foi aprovado pelo Comitê de Ética em Pesquisa do Hospital Metropolitano Odilon Behrens, sob parecer 4.480.975 e CAAE 40438420.5.0000.51290. Todos os pais ou responsáveis assinaram o Termo de Consentimento Livre e Esclarecido.

Os critérios de inclusão utilizados foram: nascimento a termo (acima de 37 semanas de idade gestacional e abaixo de 42 semanas); apresentar alteração no frênulo lingual diagnosticada pelo Protocolo de Avaliação do Frênulo da Língua em Bebês^(11,17,18)^ e Protocolo Bristol de Avaliação da Língua^(19)^; estar internado nos setores UCINCo, UCINCa ou alojamento conjunto; e não apresentar alterações craniofaciais, neuromotoras, clínicas ou respiratórias. Já para os critérios de exclusão, consideraram-se RNs que não completaram a avaliação ou que tiveram intercorrências na cirurgia.

Os participantes foram submetidos aos procedimentos de avaliação explanados a seguir.

### Caracterização da amostra (análise de prontuários)

Foi realizada a coleta de dados de registros de prontuários hospitalares, contemplando informações referentes à investigação da história pregressa dos participantes da pesquisa: idade gestacional (IG), sexo e peso na data da avaliação.

### Avaliação e diagnóstico do frênulo lingual

Foram aplicados, por uma mesma fonoaudióloga, o Protocolo de Avaliação do Frênulo da Língua em Bebês^(11,17,18)^ e Protocolo Bristol de Avaliação da Língua^(19)^.

O Protocolo de Avaliação do Frênulo da Língua em Bebês^(11,17,18)^ incluiu a avaliação da postura de lábios em repouso, a tendência do posicionamento da língua durante o choro, a forma da ponta da língua quando elevada durante o choro, a espessura do frênulo, a fixação do frênulo na face sublingual (ventral) da língua e a fixação do frênulo no assoalho da boca. Nesta pesquisa utilizou-se apenas a avaliação anatomofuncional (parte I) do protocolo, também validada como triagem^(17)^. A pontuação total pode variar de 0 (melhor resultado) a 12 (pior resultado), sendo considerado alterado o frênulo com escore igual ou maior que 7.

O Protocolo *Bristol Tongue Assessment Tool* (BTAT)^(19)^ avalia quatro aspectos do frênulo: ponta da língua; fixação do frênulo ao alvéolo inferior; elevação da língua durante o choro e de boca aberta; e protrusão da língua sobre a gengiva. As pontuações dos quatro itens são somadas e podem variar de 0 a 8, sendo considerado alterado o frênulo com escore de 0 a 3.

### Avaliação clínica da sucção não-nutritiva

A avaliação clínica da SNN foi realizada por meio da técnica de dedo enluvado e com o bebê posicionado, em supino (barriga para cima), com a região cervical e cabeça apoiadas e elevadas em relação ao restante do corpo. O dedo mínimo enluvado do pesquisador foi introduzido entre os lábios do bebê, com o dorso para cima, mediante o toque na parte anterior da língua, gengivas e palato duro e mantido na cavidade oral do bebê por um período de 3 a 5 minutos. O reflexo de sucção foi considerado presente quando o bebê respondeu com movimentos de sucção alternados com períodos de repouso. A SNN foi analisada quanto a: presença do reflexo de procura, facilidade de iniciar a sucção, vedamento labial, canolamento de língua, elevação e abaixamento de dorso de língua, elevação e abaixamento da mandíbula, coordenação dos movimentos de lábios, língua e mandíbula, força de sucção, ritmo de sucção, presença de mordida, excursões exageradas de mandíbula e sinais de estresse, conforme determinado pelo Protocolo da Avaliação da Sucção Não-Nutritiva^(20)^, idealizado para bebês prematuros. O protocolo apresenta escores para cada item avaliado, sendo que o bebê avaliado pode receber pontuação de 21 até 86. Quanto maior o escore, melhor o desempenho do bebê na SNN.

### Avaliação instrumental da sucção não-nutritiva

O equipamento utilizado neste estudo foi desenvolvido por pesquisadores do Grupo de Pesquisa em Engenharia Biomecânica da UFMG^(10)^. O método desenvolvido simula a avaliação clínica da SNN de recém-nascidos realizada na prática fonoaudiológica. O aparelho é constituído por duas partes diferenciadas, conectadas entre si por uma sonda flexível e não colabável, sendo: ponta probatória – formada por três elementos: corpo central, intermediário e cápsula de selagem. O corpo central, constituído de material plástico atóxico, é a parte encarregada de conectar o sensor (por meio do tubo de polietileno) com as partes que estarão em contato direto com o RN e permitirão captar a sucção. O intermediário é o elemento com contato direto com a boca do RN. É de silicone, atóxico, de uso individual, descartável, sendo trocado a cada participante. A cápsula de selagem é uma peça de silicone, atóxico que fixa o intermediário ao corpo central de forma que o ar não escape. O instrumento utiliza um sensor de vácuo que capta a pressão negativa e gera um sinal que, devidamente tratado, é transmitido, processado e armazenado em meio digital. Os resultados são visualizados em tempo real por meio de um computador. Para análise dos registros, adotou-se tratamento computadorizado para sistematizar as variáveis, sendo: filtro de frequência de 4,5 Hz, curva auxiliar para a identificação dos picos, início e fim dos grupos de sucção; determinação da média dos limiares e critério de pausa mínimo de 3 s.

Para avaliação, a ponta probatória foi introduzida entre os lábios do RN, mediante o toque na parte anterior da língua, gengivas e palato duro, viabilizando a captação da pressão de sucção (Figura 1). Foram realizadas duas medições para cada RN, com tempo de dois minutos para cada teste e intervalo de dois minutos entre as medições.

**Figura 1:** Figura representativa da avaliação instrumental da língua

Na caracterização das medidas objetivas do padrão de sucção desse estudo, foram definidos os seguintes parâmetros do comportamento de sucção para extração e análise:

- Número de grupos de sucção: grupos de sucção foram caracterizados pela presença de três ou mais sucções com intervalos de tempo menor que três segundos entre as sucções;
- Número de sucções: número de sucções do traçado que integram grupos de sucção;
- Tempo médio de pausas: média dos intervalos de tempo de realização das pausas;
- Valor de pressão média: média dos picos de pressão dos grupos (kPa).

Todos os seis RNs foram submetidos ao procedimento de frenotomia lingual realizado por uma mesma equipe da cirurgia pediátrica, composta por três cirurgiões. O procedimento foi realizado com tesoura, seguindo o mesmo protocolo, para todos os casos. As avaliações clínica e instrumental da sucção foram realizadas antes da frenotomia e até 48h após o procedimento, a clínica seguida da instrumental, sendo o tempo médio de coleta cerca de 20 minutos.

### Análise dos dados

Foi realizada análise descritiva da distribuição de frequência de todas as variáveis categóricas e análise das medidas de tendência central e de dispersão das variáveis contínuas. Também foi realizada a análise de comparação entre as variáveis de exposição em estudo e os eventos, utilizando os testes T de Student pareado para amostras dependentes com distribuição normal e teste de Wilcoxon para amostras dependentes com distribuição não normal, sendo aplicação do teste de Shapiro-Wilk para definição da normalidade dos dados. Para correlacionar as variáveis apresentadas no estudo, utilizou-se o teste de Friedman. Consideraram-se como associações estatisticamente significantes os resultados que apresentaram nível de significância de 5%, e, para entrada, processamento e análise dos dados quantitativos foi utilizado o programa *Statistical Package for Social Sciences* (SPSS), versão 25.0.

## DISCUSSÃO

A Tabela 1 apresenta informações sobre as características dos RNs no que se refere à idade gestacional, peso, setores de internação e escores dos protocolos de avaliação do frênulo. Após a realização do procedimento cirúrgico, houve diminuição do escore no Protocolo de Avaliação do Frênulo da Língua em Bebês e aumento dos escores no Protocolo Bristol de Avaliação da Língua. A média de pontuação da avaliação clínica da SNN pré-frenotomia foi de 39,8 nos itens positivos e 15,6 nos itens negativos e, pós-frenotomia foi de 77,3 nos itens positivos e 3,1 nos itens negativos.

**Tabela 1:** Medidas descritivas das variáveis da amostra

**Variáveis Explicativas**		**n**	**%**	**µ (±DP)**
**Idade Gestacional**	37 semanas	3	50	39,3 (**±**3,6)
	38 semanas	1	16,7	
	40 semanas	2	33,3	
**Peso**	≥1.500 g e ≤2.500 g	3	50	2.658,33 (**±**587,97)
	>2.500 g e ≤3.000 g	0	0	
	>3.000 g	3	50	
**Escore da Avaliação do Frênulo lingual**	Protocolo Bristol – Pré-frenotomia	6	100	3 (**±**2,4)
	Protocolo Bristol – Pós-frenotomia	6	100	6,3 (**±**0,8)
	Protocolo de Avaliação do Frênulo da Língua em Bebês – Pré-frenotomia	6	100	8 (**±**1,3)
	Protocolo de Avaliação do Frênulo da Língua em Bebês – Pós-frenotomia	6	100	4,2 (**±**1,9)
**Setores**	UCINCo	2	33,3	-
	UCINCa	1	16,6	
	Alojamento Conjunto	3	50	

**Legenda:** n = número de participantes; % = porcentagem válida; µ = média; DP = desvio padrão; UCINCo = Unidade de Cuidados Intermediários Convencional; UCINCa = Unidade de Cuidado Intermediário Neonatal Canguru.

As Figuras 2 e 3 apresentam os traçados da avaliação instrumental da SNN dos seis RNs. É possível verificar aumento significativo da quantidade de sucções, assim como da quantidade de grupos, além de aumento da pressão média.

**Figura 2:** Gráficos da avaliação instrumental da sucção não nutritiva antes (A) e após (B) frenotomia – Casos 1 a 3

**Figura 3:** Gráficos da avaliação instrumental da sucção não nutritiva antes (A) e após (B) frenotomia – Casos 4 a 6

A Tabela 2 apresenta a comparação das medidas da avaliação instrumental da SNN entre os momentos antes e após frenotomia. Houve diferença com significância estatística para o número de grupos de sucções, número de sucções totais e pressão média.

**Tabela 2:** Comparação das medidas objetivas da avaliação instrumental da sucção não-nutritiva antes e após frenotomia

**Variáveis (Parâmetros)**	**n**	**Pré frenotomia (µ)**	**Pós frenotomia (µ)**	**Valor de p**
**Nº grupos de sucções**	6	7,83	12,5	0,028^1^*
**Nº sucções totais**	6	36,5	70,8	0,045^2^*
**Nº médio de sucções por grupo**	6	4,19	5,76	0,1622
**Tempo médio de pausas (s)**	6	13,95	5,18	0,0682
**Pressão média (*kPa*)**	6	-7,03	-13,78	0,006^2^*

1 Teste de Wilcoxon;

2 Teste t pareado

* Resultados estatisticamente significantes com valor de p≤0,05

**Legenda:** Nº = número; n = número de participantes; s = segundos; *kPa* = quilopascal; µ = média

A análise descritiva dos tópicos do Protocolo de Avaliação da Sucção não-nutritiva revelou que todos os recém-nascidos apresentaram reflexo de procura e iniciaram facilmente a sucção, independente da alteração no frênulo lingual. Contudo, todos os outros aspectos sofreram interferência, fato observado pela comparação dos itens positivos (que aumentaram) e negativos (que diminuíram) antes e após frenotomia no grupo avaliado. É possível verificar associações estatisticamente significativas em dez dos 12 itens, sendo: vedamento labial, canolamento lingual, elevação e abaixamento de dorso de língua, elevação e abaixamento de mandíbula, coordenação de movimentos de lábios, língua e mandíbula, força de sucção, ritmo de sucção, mordidas, excursões exageradas de mandíbula e sinais de estresse (Tabela 3).

**Tabela 3:** Comparação dos parâmetros da avaliação clínica da sucção não-nutritiva das amostras pareadas antes e após frenotomia

**Variáveis**		n	**Pré frenotomia (µ)**	**Pós frenotomia (µ)**	**Valor de p**
POSITIVOS	Reflexo de procura	6	4	4	0
	Sucção iniciada facilmente	6	2,67	4	0,3461
	Vedamento labial	6	4.83	8,83	0,039^2^*
	Canolamento	6	3	7,5	0,031^1^*
	Elevação e abaixamento de dorso de língua	6	4,5	7,5	0,012^2^*
	Elevação e abaixamento de mandíbula	6	4,83	7,5	0,048^1^*
	Coordenação dos movimentos de lábios, língua e mandíbula	6	6,5	12,5	0,026^1^*
	Força de sucção	6	4,67	10	0,031^1^*
	Ritmo de sucção	6	3,33	9,67	0,008^2^*
NEGATIVOS	Mordidas	6	-2,16	-0,33	0,002^2^*
	Excursões exageradas de mandíbula	6	-2,66	0,66	0,001^2^*
	Sinais de estresse	6	-10,83	-2,5	0,034^1^*

1 Teste de Wilcoxon;

2 Teste t pareado

* Resultados estatisticamente significantes com valor de p≤0,05

**Legenda:** n = número de participantes; µ = média

O principal achado da presente pesquisa é que após a frenotomia lingual houve melhora significativa nos parâmetros clínicos e instrumentais da SNN.

A SNN típica ocorre a uma taxa de duas sucções por segundo, sendo duas vezes mais frequente que a SN^(6)^. Em um estudo norte-americano^(21)^, realizou-se uma avaliação instrumental, chamada *NeoSuck Assessment of the NTrainer System* para quantificar fatores relacionados ao desempenho da SNN. Foram avaliados grupos de sucções por minuto, número de sucções em um minuto e pressão de pico durante a sucção^(21)^, corroborando os achados do presente estudo, já que se encontrou um número médio de sucções por grupo entre 4 e 6.

Na literatura^(11,16)^ é dito que alterações do frênulo lingual podem impactar a sucção. Este fato foi evidenciado no presente estudo, visto que, após frenotomia, os pacientes apresentaram melhor organização e distribuição dos grupos de sucção, assim como, maior amplitude do sinal, mostrando aumento da pressão de sucção.

Utilizando-se um bico artificial com pequenos sensores instalados, pesquisadores^(22)^ mediram a força aplicada no mamilo pela língua de lactentes saudáveis e com dificuldade de sucção devido à anquiloglossia. Houve diferença estatisticamente significativa entre a força máxima medida na ponta e base do mamilo e a diferença de tempo necessária para atingir as forças máximas nos grupos sem e com alteração. Resultados semelhantes foram encontrados nesta pesquisa, em que se observou aumento da pressão média após frenotomia, cerca de 5 kPa, e, embora não se tenha tido um resultado estatisticamente significante, o tempo médio de pausas diminui após o procedimento, contribuindo para melhor ritmo de sucção.

Em outro estudo^(23)^, encontrou-se menor atividade elétrica dos músculos supra-hioideos durante a sucção das mamas em lactentes com o frênulo fixado no ápice da língua, independentemente da idade. Esse resultado indica que características anatômicas do frênulo lingual podem reduzir a atividade muscular, devido à restrição do movimento da ponta da língua durante a sucção, e corroboram o fato de que o frênulo alterado tem impacto na atividade da musculatura supra-hioidea, criando movimentos compensatórios. Na presente pesquisa, verificou-se que a elevação e o abaixamento de mandíbula, excursão mandibular exagerada, mordidas e sinais de estresse melhoraram ou desapareceram após a frenotomia, melhorando o padrão de sucção dos recém-nascidos e eliminando movimentos faciais e mandibulares compensatórios na avaliação clínica da SNN.

A avaliação do frênulo lingual é obrigatória^(11)^ e faz parte do exame físico do recém-nascido, porém há controvérsias entre os profissionais de saúde quanto à classificação do frênulo lingual alterado. A maioria utiliza critérios subjetivos, correlacionados com a clínica, para o diagnóstico da anquiloglossia. O uso de um protocolo clínico de avaliação do frênulo lingual é necessário para auxiliar o profissional a estabelecer o correto diagnóstico e prescrever o respectivo tratamento.

Recentemente, o Ministério da Saúde recomendou a utilização do Protocolo Bristol (*Bristol Tongue Assessment Tool*) para profissionais capacitados da equipe de saúde^(19)^ e o uso do Protocolo de Avaliação do Frênulo da Língua em Bebês é indicado e preconizado pela lei federal para a utilização no exame do Teste da Linguinha, obrigatório nas maternidades e hospitais, desde 2014. Uma vez que, na prática clínica, cada serviço utiliza o protocolo mais adaptável e apropriado ao cenário, optou-se por aplicar ambos na presente pesquisa.

Observou-se nesse estudo que os escores dos protocolos utilizados para mensuração modificaram-se após a frenotomia, mostrando ganho funcional e anatômico da mobilidade lingual. A liberação cirúrgica da anquiloglossia por meio da frenotomia pode corrigir a restrição do movimento da língua durante a alimentação para permitir uma amamentação mais eficaz e diminuir a dor mamilar materna resultante da diminuição do atrito entre a gengiva/língua inferior da criança e o mamilo^(24)^. No presente estudo, antes da frenotomia, todos os RNs apresentavam dificuldade de manter a pega e, em três casos (Casos 1, 4 e 6), as mães apresentavam fissuras mamilares.

Em um estudo^(25)^, cujo objetivo foi examinar a influência da frenotomia em recém-nascidos com menos de 12 semanas com anquiloglossia posterior, quantificando as mudanças na amamentação e na dor mamilar materna por meio do instrumento LATCH, foi observado que houve melhora significativa na pontuação LATCH imediatamente após a frenotomia. Neste estudo, não se avaliou a funcionalidade e efetividade da frenotomia durante a amamentação, contudo, os dados encontrados na avaliação objetiva da SNN sugerem que a frenotomia trouxe benefícios para o padrão de sucção desses recém-nascidos.

De acordo com os resultados obtidos no estudo de Martinelli et al.^(26)^, após frenotomia em bebês com anquiloglossia, houve aumento do número de sucções e diminuição da duração das pausas entre os grupos de SN, além de redução nas queixas das mães a respeito de: cansaço do bebê ao mamar, longas pausas entre as sucções, curto intervalo entre as mamadas, períodos curtos de sono entre as mamadas, padrão mordedor do mamilo, ruídos durante a amamentação, dor no mamilo, regurgitação e tosse. O presente estudo, analisou apenas a SNN, mas também evidenciou melhora do padrão de sucção após frenotomia, visto que, após o procedimento, houve aumento médio de aproximadamente cinco grupos de sucções e de 35 sucções durante o tempo avaliado, aumento da pressão média em cerca de 6 kPa e o tempo médio de pausas diminui em torno de nove segundos. Já na avaliação clínica da SNN, verificou-se que a força de sucção aumentou cerca de cinco pontos, o ritmo melhorou e, em relação aos pontos negativos, as pontuações referentes à presença de mordidas e sinais de estresse diminuíram aproximadamente dois e oito pontos, respectivamente.

Sugere-se que futuras pesquisas realizem o acompanhamento a longo prazo dos participantes e incluam avaliação da SN, a fim de verificar os preditores e efeito da intervenção na resolução de possíveis dificuldades de amamentação. O número reduzido de participantes e a ausência de cegamento do avaliador constituem limitações do presente estudo. No entanto, este é um estudo pioneiro na apresentação de medidas quantitativas de sucção não-nutritiva e análise comparativa dessas medidas nos indivíduos antes e após frenotomia.

## CONSIDERAÇÕES FINAIS

Este estudo mostrou melhora considerável do padrão de sucção não-nutritiva em bebês submetidos à frenotomia lingual, considerando as medidas instrumentais (número de grupos de sucções, número de sucções totais e pressão média) e clínicas (vedamento labial, canolamento, elevação e abaixamento de dorso de língua, elevação e abaixamento de mandíbula, força de sucção, ritmo de sucção, mordidas, excursões exageradas de mandíbula e sinais de estresse), com efeitos favoráveis na funcionalidade dos movimentos linguais.
